# Crystal structures of PI3K-C2α PX domain indicate conformational change associated with ligand binding

**DOI:** 10.1186/1472-6807-8-13

**Published:** 2008-02-29

**Authors:** Gary N Parkinson, David Vines, Paul C Driscoll, Snezana Djordjevic

**Affiliations:** 1 Cancer Research UK Biomolecular Structure Group, The School of Pharmacy, University of London, 29-39 Brunswick Square, London WC1N 1AX, UK; 2 The Ludwig Institute for Cancer Research, c/o Structural and Molecular Biology Research Department, University College London, Gower Street, London WC1E 6BT, UK; 3 The Institute of Structural Molecular Biology, Structural and Molecular Biology Research Department, University College London, Gower Street, London, WC1E 6BT, UK

## Abstract

**Background:**

PX domains have specialized protein structures involved in binding of phosphoinositides (PIs). Through binding to the various PIs PX domains provide site-specific membrane signals to modulate the intracellular localisation and biological activity of effector proteins. Several crystal structures of these domains are now available from a variety of proteins. All PX domains contain a canonical core structure with main differences exhibited within the loop regions forming the phosphoinositide binding pockets. It is within these areas that the molecular basis for ligand specificity originates.

**Results:**

We now report two new structures of PI3K-C2α PX domain that crystallised in a P3_1_21 space group. The two structures, refined to 2.1 Å and 2.5 Å, exhibit significantly different conformations of the phosphoinositide-binding loops. Unexpectedly, in one of the structures, we have detected a putative-ligand trapped in the binding site during the process of protein purification and crystallisation.

**Conclusion:**

The two structures reported here provide a more complete description of the phosphoinositide binding region compared to the previously reported 2.6 Å crystal structure of human PI3K-C2α PX where this region was highly disordered. The structures enabled us to further analyse PI specificity and to postulate that the observed conformational change could be related to ligand-binding.

## Background

Many cellular processes such as growth, signalling, cytoskeletal rearrangement and membrane trafficking depend upon lipid-protein interactions [1,2]. Proteins involved in these processes commonly contain one or more of specialised lipid-binding protein domains such as FYVE [3], PH [4,5], FERM [6], C2 [7], PX [8] and others. PX domains have first been described in p40^phox ^and p47^phox ^subunits of NADPH oxidase [9]. Since then they have been identified in a variety of proteins that are involved in cell signalling and membrane trafficking. The involvement of PX domains in these cellular processes stems from their capacity to bind phosphoinositides that are not only integral components of many cellular membranes but are also utilized as secondary messengers [1,2]. Phosphoinositides (PIs) form a class of membrane phospholipids whose basic structure is termed phosphatidylinositol (PtdIns). PtdIns consists of a diacylglycerol linked by a phosphodiester bond to position 1 of an inositol head group. Other phosphoinositides are formed by phosphorylation of the inositol group at position 3, 4 or 5 or combination of these sites. These 3, 4, and/or 5- phosphorylated PtdIns derivatives are metabolized by the specific lipid phosphatases generating a dynamic pool of variously phosphorylated PtdIns species in the cell [10]. Protein-binding to the specific PIs, via a lipid-binding domain, not only directs the subcellular localisation of that protein but crucially it can also affect its biological activity [11-13].

PX domain-harbouring proteins exhibit different lipid binding specificities. For example, it was reported that Vam7p [14], sorting nexin 3 [15] and p40^phox ^[16,17] bind PtdIns3P. P47^phox ^exhibits specificity for PtdIns(3,4)P_2 _[16], yeast protein Bem1p binds PtdIns4P [18], while the PX domain of PI3K-C2α preferentially binds PtdIns(4,5)P_2 _[19]. The structural basis for this property that PX domains differentiate between PIs, together with functional implications of their lipid-binding specificities, has led to a great deal of interest. The first insight into the lipid-binding mode of PX domains came from the structure of p40^phox ^in complex with PtdIns3P [20]. The structure revealed specific basic residues that are involved in interactions with phosphate groups at the inositol positions 1 and 3. Further structure reports of other PtdIns3P-binding proteins have shown that analogous residues were also present in their PX domains [21,22]. The crystal structure of p47^phox ^revealed a phosphatidic acid site in addition to the main PtdIns(3,4)P_2 _binding region [23]. More recently, the structures of yeast Bem1p [24] and human PI3K-C2α [25] PX domains, two domains that do not bind 3-phosphorylated PtdIns, have been reported addressing the origin of their lipid-binding specificity. These two reports were elegantly complemented by membrane binding analyses providing a strong link between detailed structural and functional characteristics of PX domains.

We now report two structures of the PI3K-C2α PX domain that were crystallised in a new crystal form, P3_1_21, and refined to 2.1 Å and 2.5 Å respectively. The two structures provide further insight into the possible mode of PtdIns(4,5)P_2 _binding. The family of PI 3-kinases (PI3Ks) catalyses phosphorylation of the cytosol-oriented inositol head group at the 3-hydroxyl position [26,27]. Four known lipid products that act as secondary messengers are generated by different classes of PI 3-kinases. It has been demonstrated that the *in vitro *substrates of Class II PI 3-kinases, comprising PI3K-C2α, PI3K-C2β and PI3K-C2γ, are PtdIns and PtdIns4P [28,29]. While all PI3Ks must interact with phosphoinositides as phosphorylation substrates within the catalytic domain active site, membrane-associated class II PI3Ks are unique in that they additionally contain PX and C2 domains appended to the C-terminus of the kinase domain [30]. Curiously, deletion of either the C-terminal C2 domain or the PX domain from PI3K-C2α does not affect the subcellular localisation of this enzyme and the cellular function of these apparent structural embellishments remains unresolved [31].

Class II PI3Ks are activated by the range of stimuli such as chemokines [32], cytokines [33] and growth factors [34]. PI3K-C2α specifically can be activated by clathrin, regulating clathrin assembly [35] and clathrin-mediated membrane trafficking [36]. This enzyme was also identified as required for ATP-dependent priming of neurosecretory granule exocytosis [37] and as vital in vascular smooth-muscle contraction [38]. Recently, it was demonstrated that PI3K-C2α contributes to insulin-induced translocation of the glucose transporter GLUT4 to the plasma membrane, underlining a definitive role of PI3K-C2α in insulin signalling [39]. Furthermore PI3K-C2α also translocates to the plasma membrane upon insulin stimulation [39]. The two structures of PI3K-C2α PX domain described here provide a more complete picture of PtdIns(4,5)P_2 _binding-site and allow interpretation of some of the available mutagenesis data describing the membrane penetration properties of PI3K-C2α PX domain as reported by Stahelin et al [25]. In addition, the significant variation in the conformation of the PI-binding loops in the two structures, due to a putative ligand found in one of the PtdIns(4,5)P_2 _binding-sites, demonstrates explicitly the intrinsic conformational plasticity of this protein module in the region of its ligand interaction site.

## Results

### Overall structure description

Spontaneously formed crystals of PI3K-C2α PX domain belong to P3_1_21 space group with cell dimensions of a = b = 56.9 Å and c = 93.0 Å, containing one molecule of the PX domain in the asymmetric unit. The molecule packs in a very different fashion when compared to previously reported structure that crystallised in space group P 4 3 2 (a= 116 Å). In both crystal forms, symmetrically related molecules do not form extensive surface residue contacts (Vm = 3.2 Å ^3^/Da in P3_1_21 cell). The conformations of the two loops that are implicated in ligand binding (discussed below) appear to not be affected by crystal packing with exception of the β1-β2 turn (residues 1434–1438) which in our higher resolution structure comes in close contact with symmetrically related residues from the neighbouring molecule.

**Figure 1:**
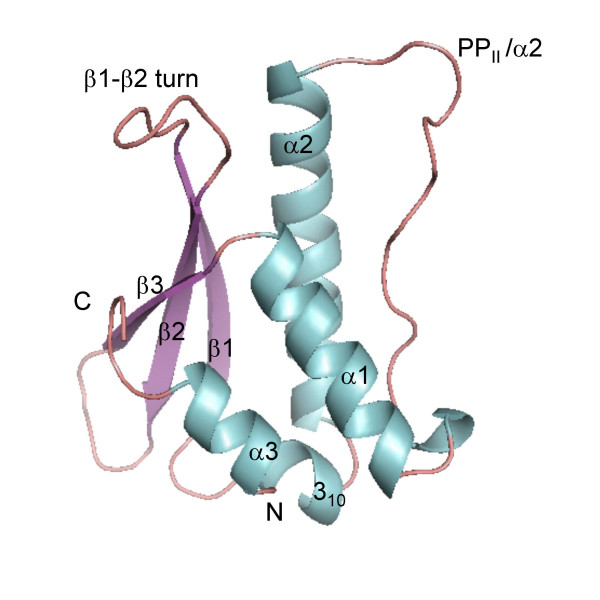
Cartoon diagram of PI3K-C2α PX domain. The colour scheme is based on the secondary structure with helices shown in blue and β-strands in purple.

The PI3K-C2α PX domain folds into the canonical PX polypeptide chain topology comprising a three-stranded β-sheet meander that packs against a subdomain of 3 α-helices (Figure 1). Distinct features of this domain are the PI binding/membrane penetrating loop (residues 1488–1497) and β1-β2 turn which also contributes to formation of the PI binding site. The membrane penetrating loop is sometimes referred to as the PP_II_/α2 loop, as this region is preceded by a type-II poly-proline helix. We solved the structure of PI3K-C2α PX domain by molecular replacement using the human P47^phox ^PX domain structure as a model template, as at that time the structure by Stahelin et al was not available. Our search model did not include any of the loops connecting the secondary structure elements as these belong to the regions of poor sequence similarity with the template model. Overall, the PX domain of PI3K-C2α shares 25 % sequence identity with human P47^phox ^of NADPH oxidase. As the refinement of the replacement solutions proceeded we were able to build the residues that were missing in the starting model. Particularly challenging was building the α1-α2 connection as this region has weak sequence homology and is 6 residues shorter in PI3K-C2α PX domain compared to P47^phox^.

From the first electron density maps it became apparent that electron density at the side chain for residue 1450 did not correspond to that of tryptophan as expected based on the predicted amino acid sequence. In fact the electron density strongly suggested that the side chain is that of an arginine. At that stage we sequenced the full length of the several clones of the DNA construct used for the preparation of the protein and confirmed that indeed that residue was arginine. In addition residue 1464 was found to be an aspartate in place of the anticipated valine based on the human sequence NP_002636, GI:4505799; this side chain fitted much better in the electron density maps. The discrepancy with reported protein sequence NP_002636 was also noted by Stahelin et al [25] which commented on a possible data base error. Interestingly, the amino acid sequence that fits best with the data is 100 % identical to the PI3K-C2α sequence of Pongo pygmaeus, GI:73921535. On the other hand the DNA sequence is an exact match to human cDNA clone MGC:142218 IMAGE:8322710 and in the most recent ENSEMBL entry (Oct 2007) this discrepancy was corrected with the reference to SNPS annotation as variable positions refSNP ID: rs1065446 and refSNP ID: rs1143107. The capacity to recognize a different side chain in our initial electron-density map was taken as a validation of our molecular replacement solution.

The model obtained based on the 2.5 Å data contains coordinates for residues 1421–1532 of the domain and an additional GSHH tag-derived sequence at the C-terminus. Residues 1490, 1491 and 1493–1496 were modelled as alanines since their side-chains were not clearly visible in the electron-density maps. This model, which we refer to here as 'structure B', was refined to an R_factor_/R_free _of 0.231/0.311. Structure A was obtained from the 2.1 Å data set and unlike in structure B we were unable to determine the positions of the main chain atoms for residues 1490, 1491 and 1492; these atoms were not included in the final model. This final model additionally contained six glycerol molecules and 92 water molecules and is refined to an R_factor_/R_free _of 0.235/0.280.

### Comparison with other PX domain structures

**Figure 2:**
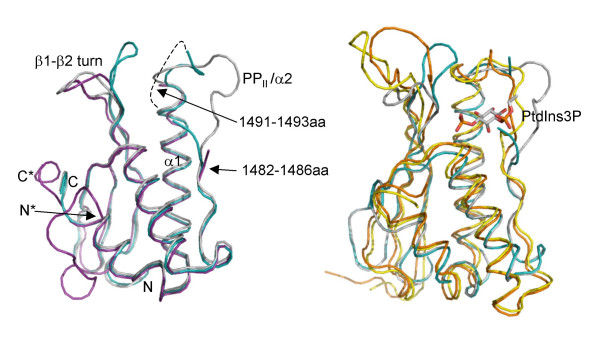
C alpha traces of structures A (cyan) and B (silver). a) The two structures were superimposed with the Cα trace of 2IWL [25] PI3K-C2α structure (magenta). Dashed line represents most likely positions for the three residues disordered and not observed in structure A. N and C denote the positions of the N- and C-termini of the structures A and B, while N* and C* point to the N- and C- terminal ends of structure 2IWL. b) Structures A and B were superimposed with the Cα trace of 1OCU (orange), the yeast PX domain protein Grd19p and 1OCS (yellow), Grd19p protein in complex with phosphatidylinositol-3-phosphate. The phosphatidylinositol-3-phosphate is drawn as sticks coloured on atom type.

The core secondary structure elements superimpose closely (Cα RMSD = 0.61 Å) with previously reported PI3K-C2α PX structure (2IWL) which was determined by SIR methods. While previously the regions of the domain involved in PI binding were disordered, the two structures that we have obtained provide a more complete model for these parts of the molecule. Overlay of the two structures (Figure 2A) shows that the polypeptides used for the crystallisations differ: while we have used the polypeptide that spans residues 1421–1532 of the protein, 2IWL structure is of a longer polypeptide that corresponds to the region 1405–1541 (with residues 1409–1539 visible in the structure). The two polypeptides crystallised in distinctly different space groups: P 3_1 _2 1 and P 4 3 2 respectively, placing the two molecules in very different packing environments. The longer N-terminus, which does not comprise a part of a canonical globular PX domain structure, cannot be accommodated within the unit cell that describes the structures that we report. Similarly, the packing arrangement in P 4 3 2 symmetry equivalent to that of 2IWL structure cannot accommodate the conformations of the phosphoinositide-binding loops that we observe.

Our two new structures, A and B, are however not identical: they have two significantly different conformations of the putative PI binding region. Structure B has a more open PI binding site with the PP_II_/α2 loop and β1-β2 turn presenting conformations similar to that previously seen in the yeast PX domain protein Grd19p. On the other hand, the higher resolution structure, structure A, exhibits a conformation that gives a more closed appearance of the PI binding cavity, as if the PP_II_/α2 loop has collapsed to close the binding site. The conformations of the two binding sites and the comparison with the Grd19p protein are shown in Figure 2. Although the PP_II_/α2 loop in the higher resolution structure still contains significant disorder sufficient residues are visible in the structure to provide a clear indication of the overall shape of the binding site. The main area where the two loop conformations diverge is after the PGFP sequence (residues 1483–1486) giving an open or closed loop arrangement depending on the backbone torsion angles of the following residues. The conformational difference cannot be described by a hinge motion as the main parts of the two loops could not be closely superimposed. The overall appearance of the PI binding cavity is also affected by the differing conformations of the β1-β2 loop with the largest Cα displacement of ~13 Å compared to ~14 Å for the PP_II_/α2 loop. As shown in Figure 2b, the closed conformation of structure A is more similar to the PtdIns3P-bound Grd19p structure, while the PI3K-C2α PX structure B is closer to the un-liganded Grd19p structure. Two different conformational states of Grd19p have been attributed to the ligand-binding and our two PI3K-C2α PX domain structures suggest that binding of PI ligands to the PI3K-C2α PX domain might also be accompanied by conformational change.

### Putative PI-binding site

**Figure 3:**
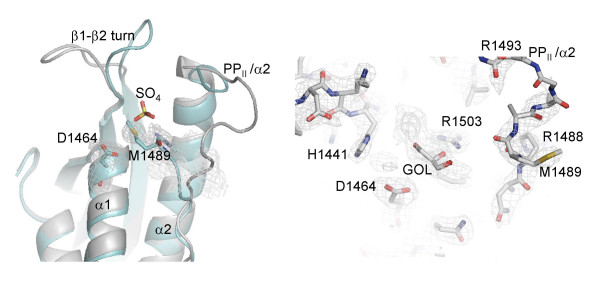
PI binding pocket. a) Ribbon representation of the structures A (cyan) and B (silver) with the (2Fo-Fc) electron density for the side chain of Met1489 residue in structure A. The position of a bound-sulphate in the 2IWL structure is depicted for comparison. b) View of ligand-binding pocket in structure B, showing glycerol modelled into 2Fo-Fc electron density that is central to the proposed phosphoinositide binding site. Structure B is shown as a stick model and coloured based on atom types.

Interestingly, in the 'collapsed' form observed in structure A, the equivalent side chain of the Met1489 folds inside the cavity with its sulphur atom positioned near the sulphate binding site of the 2IWL structure (Figure 3a). In addition, the shape of the Met side chain closely resembles one half of the inositol ring when modelled at that site. In contrast, in the 'open' conformation of the loop from structure B, the Met side chain protrudes out of the lipid binding site. However, unaccounted electron density was clearly visible inside the cavity of the putative PI-binding area linking to residue Arg1503 (Figure 3b). The density has a relatively flat shape and for the purpose of illustration we have positioned a glycerol molecule within the density in Figure 3b. We have attempted to model this density as maleic acid, glucose, glycerol and inositol, none of which adequately describes the density and all of which had only a minor impact on R_free_. Presumably, an unidentified component of the buffer or LB media was trapped in the binding site of the PX domain.

**Figure 4:**
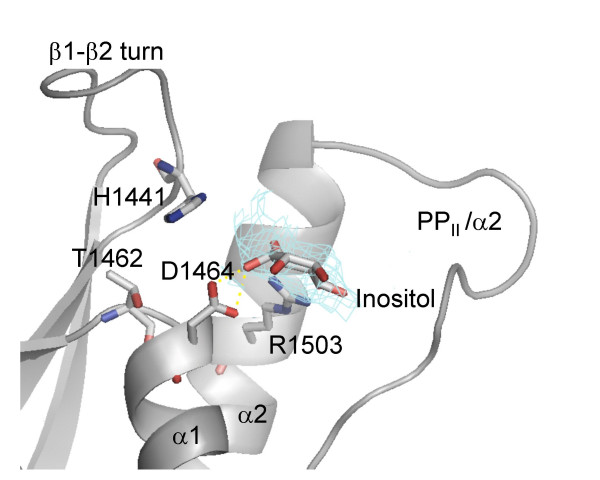
Model of an inositol molecule in the proposed PI-binding site in structure B. Inositol was positioned into 2Fo-Fc electron density. Contacts to Asp1464 residue and Arg1503 residue are highlighted.

The putative ligand found in the B structure delineates the most likely ligand interaction site, and in Figure 4 we have positioned an inositol ring to overlap with the density in order to further discuss aspects of PI binding site. Comparison of the PI3K-C2α structure in this region with P40^phox ^or Grd19p, both of which bind 3-phosphorylated PIs, immediately reveals that the conserved basic residues involved in the specific interaction with the 3-phosphate group are missing in the PI3K-C2α PX domain. Thus the two structures reported here reinforce the observation made by Stahelin et al, that there is a loss of positive selection for the phosphoryl-appendage at the position 3 of the PtdIns. Namely, in place of residue Arg58 in P40^phox ^and equivalent Arg residues in P47^phox ^and Grd19p that are engaged in a specific interaction with the 3-phosphoryl group there is residue Thr1462 in PI3K-C2α PX domain structure. In addition, p40^phox ^residue Arg60, which is implicated in interaction with the 1-phosphate in PtdIns3P-bound structure, corresponds to residue Asp1464 in the PI3K-C2α PX domain. It was suggested by Stahelin et al that Asp1464, a residue that is conserved in all PI3K-C2α sequences, imposes negative selection against 3-phosphorylated PtdIns species. Based on our model of the inositol ring in putative ligand electron density we would suggest that this residue not only is incompatible with phosphate binding but that it might be involved in specific interaction with the 3-OH and/or 2-OH groups of PtdIns(4,5)P_2 _ligand. A similar type of interaction is present in many ribose-containing coenzyme binding sites where acidic side chain serves the function of discriminating between NADH and NADPH molecules [40-42]. A specific interaction with 3(2)-OH group(s) would orient PtdIns(4,5)P_2 _such that the phosphoryl groups would be positioned for the appropriate interactions with the residues of the PP_II_/α2 loop. In a recently reported structure of the PX domain of Bem1p, which is PtdIns4P-specific, the equivalent position is occupied by a Gln residue. However, this PX domain, while highly selective for PtdIns4P, exhibited only modest affinity for PtdIns4P-containing vesicles when compared to the apparent affinity of PI3K-C2α PX domain for its cognate PI.

In structure B, the putative ligand appears to form hydrogen bonds with Arg1503, a highly conserved residue in PX domains. Modelling of an inositol ring in this site (Figure 4) shows that, with minor adjustments of torsion angles Arg1503 would be optimally posed to interact with a phosphoryl group at inositol position 4 and that another residue from PP_II_/α2 loop would likely be involved in interaction with the 5-phosphate.

## Discussion

### Variant PP_II_/α2 loop conformational states and implications for PI binding

Although the two structures reported here provide a more complete model of the PI3K-C2α PX domain, we are still unable to give a detailed atomic description of the PI binding-site. However, the discrete conformational states of the PP_II_/α2 loop and β1-β2 turn in our two structures, as well as the presence of a bound ligand in one of them, allow us to postulate that PI binding is associated with conformational changes. A similar observation was made for the PX domain of Grd19p although in this case a more 'closed' conformation was associated with the ligand-bound form of the protein. To date no structures of a 4- or 5- phosphorylated PtdIns molecule bound to the binding site of the corresponding PX proteins have been determined and hence the precise molecular basis of the PI specificity of these molecules remains unresolved. It is most likely that the conserved residue Arg1503 is engaged in the interaction with phosphoryl groups at these sites even though the same residue is engaged in interaction with non-substituted OH groups in those molecules that exhibit strict PtdIns3P specificity. Therefore, other contributors to the 4,5-phosphate binding specificity would have to exist. We suggest that, at least in part, this specificity arises from the intrinsic flexibility of the PP_II_/α2 loop and β1-β2 turn as shown explicitly by our two structures and implied by the 2IWL structure. This flexibility of the binding site would affectively enable the molecule to adopt a conformation required to accommodate the phosphoryl groups at PtdIns positions 4 and 5 and to still place Arg1503 residue at the right distance for effective interaction. In addition, other positively charged residues in the region would contribute to formation of the appropriate complementary electrostatic potential needed to compensate for the negative charges associated with the two phosphoryl groups. In the report by Stahelin et al, some of the residues in this region have been mutated and shown to impact the binding affinity of the domain. For example, mutation of the residue Arg1503 completely abolishes PI binding. The second most profound mutation is that of Arg1493 which results in 23- fold decrease in PtdIns(4,5)P2 binding affinity. Although we were able to model backbone atoms for this residue in one of our structures, the side chain for this residue is not visible in the electron density map. Simple modelling of the Arg torsion angles suggests that this side chain could fold into the PI binding cavity and be engaged in electrostatic interaction with 5-phosphoryl group of the ligand. Interestingly Arg1488, which when mutated to Ala leads to 7-fold decrease in PtdIns(4,5)P_2 _binding affinity, presents different conformations in our two structures. In the 'closed' structure A conformation of the PP_II_/α2 loop this side chain is oriented away from the core of the domain into the solvent region, while in the B structure with the 'open' PP_II_/α2 loop conformation, it is turned more towards the interior of the loop. This observation reinforces the notion that ligand-binding to the PI3K-C2α PX domain is associated with local conformational changes that would allow for the optimal modelling of the binding site to match the ligand regiochemistry and to complement its electrostatic potential distribution with the residues that form PI binding surface.

## Conclusion

Our two new structures of PI3K-C2α PX domain show significantly different conformations of the PI binding region. These differences are attributed to the presence of a putative ligand in the PtdIns(4,5)P_2 _binding cavity in one of the crystal structures. The ligand-bound structure exhibits an 'open' conformation of the PI binding region. In contrast, in unliganded structure the main loop of the PtdIns(4,5)P_2 _binding cavity collapses into the cavity creating a 'closed' conformation of the region. The two structures reported here together with that reported previously by Stahelin et al, provide compelling evidence for the plasticity of this domain which might be required to achieve full PtdIns(4,5)P_2 _specificity. In addition, we suggest that residue Asp1464, which is conserved in PI3K-C2α proteins, might be involved in the specific recognition of 3- OH group within PtdIns(4,5)P_2 _molecule.

## Methods

### Protein expression, Purification and Crystallisation

The full-length PI3K-C2α gene in pBKCMV vector was obtained from Prof Peter Shepherd's laboratory [43]. A portion of the full length PI3K-C2α gene corresponding to the amino acid residues 1421–1532 was amplified by PCR methods. This gene construct was inserted into a modified pET-21(b) vector which contained only NdeI and BamHI cloning sites yielding the protein product with a 6His-tag at the C-terminus together with a Gly-Ser linker (part of BamHI cloning site). This construct was initially sequenced to confirm insertion of the fragment in-frame and subsequently was resequenced (VWR sequencing services) to validate apparent amino acid discrepancies based on the X-ray electron density maps.

Protein was expressed in BL21(DE3) cells at 37°C and purified by Ni^2+^-affiinty chromatography. During the purification procedure, it was necessary to keep concentration of the protein fraction bellow 2.5 mg/ml as it was noticed that the protein was susceptible to precipitation. The purified protein was dialysed into a buffer containing 10 mM Na acetate pH 5.5, 6 mM DTT and 1 mM EDTA and concentrated to 1.25 mg/ml. Initially, the protein was prepared for NMR studies and a screen was carried out, testing the solubility of the protein in a range of organic acid buffers [44]. During this procedure it was noticed that protein precipitated or crystallized under many conditions and further screens were designed to increase the yield and size of the crystals. Final crystals were obtained by the hanging drop method where 1 μl of reservoir solution was mixed with 3–6 μl of protein solution and inverted over 1 ml of 0.1 M Na-maleate pH 6.0, 10–20 % glycerol, in the reservoir. Crystals grew overnight and achieved their final size within a few days.

### Data Collection and Structure Determination

The crystals grown in the presence of 20 % glycerol were frozen in liquid nitrogen directly prior to data collection; otherwise the crystals were transferred into a cryoprotectant solution consisting of reservoir buffer plus 20% (*v/v*) glycerol before being flash-cooled in liquid nitrogen. It was found that the crystals were heterogeneous and that, even from the same batch of protein preparation, crystals exhibited different diffraction power with maximum resolution ranging from 3.5 Å to 1.9 Å. After screening several crystals two data sets were collected and used to determine the structures reported here. The first data set at 2.5 Å was obtained in-house using a R-AXISIV image plate detector system and Osmic mirrors. Higher resolution crystal data (2.1 Å) on a different crystal were collected at the ESRF, Grenoble on beamline ID-14-4. All data was processed by DENZO and scaled by using SCALEPACK [45].

The structure was initially solved with the in-house collected data set by molecular replacement using EPMR [46]. The search model was created from the X-ray structure of the un-liganded PX subunit of p47^phox ^NADPH oxidase, PDB id 1O7K. To create a starting model, the main loops in the structure were removed and the non-conserved residues were substituted with alanines. The initial molecular replacement solution was subsequently built using TURBO [47] and refined using REFMAC [48]. 10% of the reflections were selected for cross validation during refinement. The poly-histidine tag and some residues in the loop regions were not fully visible in the electron density maps and they were either excluded from the final model or their side chains were shortened to include only Cβ atoms. The higher resolution model also included six glycerol molecules. The models, as evaluated by PROCHECK [49], show good geometry with only 1 Gly residue in L-α helical region. The data collection and refinement statistics are shown in Table 1. The structures and structure factors were deposited with the PDB with ID codes 2RED and 2REA.

**Table 1:** Crystallographic data

	Crystal 1	Crystal 2
Space group	*P*3_1_21	*P*3_1_21
Unit cell dimensions (Å, °)	*a *= 56.554, *b *= 56.554, *c *= 92.894, α = β = 90, γ = 120	*a *= 56.866, *b *= 56.866, *c *= 92.996, α = β = 90, γ = 120
*Z *(number in AU)	1	1
Beamline	ID14-4	Cu, Rotating Anode
Wavelength (Å)	0.9792	1.5418
Resolution (Å)	48-2.1 (2.27–2.10)	30-2.50 (2.57–2.5)
Unique reflections to 1.9 Å	16365	5731
Reflections used	9844	5458
Completeness	98.9 (100)	89.4 (70.9)
I/σ (I) for the data set (outer shell)	43.7 (2.9)	40 (9.0)
*R*_merge _(%) (outer shell)	0.057 (0.127)	0.028 (0.107)
*R*_cryst _(outer shell)	0.235 (0.228)	0.231 (0.214)
*R*_free _(outer shell)	0.280 (0.305)	0.318 (0.236)
R.m.s.d. 1–2 bonds (Å)	0.020	0.032
R.m.s.d. 1–3 angles (Å)	1.760	2.81

Highest resolution shell in parentheses.

*R*_merge _= ∑_hkl _| (I*hkl*) - <I(*hkl*)> |/∑ I(*hkl*).

*R*_cryst _= ∑_hkl _|| F_obs _| - | F_calc _||/∑_hkl _| F_obs _|. For *R*_free _calculation, 5% of the test set amplitudes were employed, and these were not used in refinement.

## List of abbreviations

PI3K-C2α – Phosphoinositide 3-kinase C2α. PI3K – Phosphoinositide 3-kinases. PIs – Phosphoinositides. PX – Phox homology. PtdIns(4,5)P_2 _– Phosphatidylinositol 4,5-bisphosphate. PtdIns(3,4)P_2 _– Phosphatidylinositol-3,4-bisphosphate. PtdIns3P – Phosphatidylinositol 3-phosphate. PtdIns4P – Phosphatidylinositol 4-phosphate. PtdIns – Phosphatidylinositol. FYVE – cysteine-rich domain originally found in Fab1p. YOTB, Vac1p and EEA1 proteins. PH – pleckstrin homology domain. FERM – (4.1, ezrin, radixin, and moesin) domain. C2 – Calcium/Lipid-binding domain. NADPH – Nicotinamide adenine dinucleotide phosphate. ATP – Adenosine 5'-triphosphate. DTT – Dithiothreitol. EDTA – Ethylenediamine tetraacetic acid. RMSD – Root mean square deviation. V_m _– Matthews coefficient.

## Authors' contributions

GNP carried out molecular replacement and model refinement. DV created protein expression vector, purified and crystallized the protein. PD initiated the project and edited the manuscript. SD was involved in model building and manuscript preparation. All authors read and approved the final manuscript.
